# Supplementary material for: A weighted network analysis framework for the hourglass effect—And its application in the C. elegans connectome
Source: PLoS One. 2021 Oct 27;16(10):e0249846. doi: 10.1371/journal.pone.0249846 (PMC8550382; doi:10.1371/journal.pone.0249846)
Supplement: Attachment — Submitted filename: Response to Reviewers.pdf [file pone.0249846.s001.pdf]

AUTHOR RESPONSES TO REVIEWS BY EDITORS AND REVIEWERS

We are very thankful to the editor and the reviewers for taking out the time to review and provide valuable feedback on our work. We have worked to incorporate the suggestions made in the letter for minor revision. Please find below in blue text a point-by-point response to the suggestions from both editor and the reviewers. Any references to page numbers, sections, figures, and papers refer to the new revised and marked manuscript file.

Editor's Comments

Please include the following items when submitting your revised manuscript:

A rebuttal letter that responds to each point raised by the academic editor and reviewer(s). You should upload this letter as a separate file labeled 'Response to Reviewers'.
A marked-up copy of your manuscript that highlights changes made to the original version. You should upload this as a separate file labeled 'Revised Manuscript with Track Changes'.
An unmarked version of your revised paper without tracked changes. You should upload this as a separate file labeled 'Manuscript'.

Response: We have uploaded all three documents on the portal. We have also uploaded a standalone latex file and figures in addition to these three documents.

1. Please ensure that your manuscript meets PLOS ONE's style requirements, including those for file naming.

Response: The new manuscript documents uploaded on the portal have been revised to follow the PLOS ONE format guidelines as mentioned. Please note that we have not highlighted these changes in the marked manuscript as they are associated only with the format.

2. We note that you have stated that you will provide repository information for your data at acceptance. Should your manuscript be accepted for publication, we will hold it until you provide the relevant accession numbers or DOIs necessary to access your data. If you wish to make changes to your Data Availability statement, please describe these changes in your cover letter and we will update your Data Availability statement to reflect the information you provide.

Response: We have updated the code for downloading the data and implementing the analysis in a Github repository which can be found at: https://github.com/QihangYao/weighted-hourglass . We will provide the same information on the submission portal.

3. Please review your reference list to ensure that it is complete and correct. If you have cited papers that have been retracted, please include the rationale for doing so in the manuscript text, or remove these references and replace them with relevant current references. Any changes to the reference list should be mentioned in the rebuttal letter that accompanies your revised manuscript. If you need to cite a retracted article, indicate the article's retracted status in the References list and also include a citation and full reference for the retraction notice.

Response: We have not included any retracted articles. However, based on the reviewers' comments, content updates were done to the discussion section. The new references added to the paper as part of this update are listed below:

- Mattie, H., Engø-Monsen, K., Ling, R., and Onnela, J.-P. (2018). Understanding tie strength in social networks using a local "bow tie" framework. Scientific reports, 8(1):1–9.
- Rombach, M. P., Porter, M. A., Fowler, J. H., and Mucha, P. J. (2014). Core-periphery structure in networks. SIAM Journal on Applied mathematics, 74(1):167–190
- Maesa, D. D. F., Marino, A., and Ricci, L. (2019). The bow tie structure of the bitcoin users graph. Applied Network Science, 4(1):1–22.
- Borgatti, S. P. and Everett, M. G. (2000). Models of core/periphery structures. Social networks, 21(4):375–395.
- Csermely, P., London, A., Wu, L.-Y., and Uzzi, B. (2013). Structure and dynamics of core/periphery networks. Journal of Complex Networks, 1(2):93–123
- Holme, P. (2005). Core-periphery organization of complex networks. Physical Review E, 72(4):046111

Reviewer #1:

This paper is a bio-informatics paper related to neural science. It develops a new framework for hourglass analysis that takes networks weights into account while
identifying the core nodes and the extent of hourglass effect in a given weighted network. The framework is used to study the structural connectome of the C.elegans and identify intermediate neurons that form the core of sensori-motor pathways in the organism.

There are three types of neurons in hourglass analysis on the C. elegans connectome, including sensory neuron, inter-neuron and moter neuron. The paper develops a multi-edge transformation (MET) to deal with the unweighted/weighted networks. For model details, it selects core nods with large path centrality scores and defines Hourglass score as a criteria to retrieve the best hourglass property of a network. It runs a lot of experiments with random setups and sex-wise comparison to see the hourglass effect under different scenarios.

The overall paper is well written and with essential research values. The only advice is that as the network contains multiple node neuron types and is directed. it would be better if the authors can mention some interpretations or comparisons for homogeneous/heterogeneous and (un)directed networks. This content can be addressed throughout analysis or in the future work.

Response: Thanks for your valuable feedback. As suggested, we have discussed about homogeneous and heterogeneous as well as directed vs. undirected network cases in the final paragraph of the discussion section. We have copied the updated paragraph below in italicized font:

Various methods have been used previously for identifying critical neurons in the C. elegans connectome, such as graph perturbation techniques [35] and controllability frameworks [36]. The proposed framework in our paper is applicable only to directed networks with a given set of paths from source nodes to target nodes. The network may include cycles and the nodes may be of the same or different type — the hourglass method is applicable as long as we are given a set of directed paths from source to target nodes. A more extensive comparison between the hourglass framework and other related methods, such as core-periphery or rich-club approaches [37–40] can be found in [19].

More broadly, there are many approaches to identify important nodes (or rank them) in general networks [18, 39, 41]. The hourglass analysis framework is fundamentally different from approaches that are degree-based (such as k-core decomposition), that only consider shortest paths (such as betweenness centrality), or that consider all possible paths (potentially weighted based on their length, such as Katz centrality). A comparative study of existing and extended frameworks along with an interpretation of the outcomes should be done in the near future to enhance the understanding of the nature of optimization followed by naturally occurring neuronal networks.

Reviewer #2: The authors have done a good job and addressed all the comments and suggestions. The paper seems to be in a good shape now. I recommend that the paper can be accepted for publication in the current form.

Response: Thanks for your valuable feedback on the paper.
